# Resting Heart Rate as a Physiologic Marker of Steatosis in MASLD: NHANES 2017–2020 Analysis

**DOI:** 10.3390/medicina62081480

**Published:** 2026-08-01

**Authors:** Ethan Shamsian, John Ralph Mack, Mahinaz Mohsen, Anika Makol, Sameer Rao, Rohan Karkra, Michael Bebawy, Muhammad Hassaan Arif Maan, Fariha Ilyas, Ahmed Al-Khazraji, Paul Gaglio

**Affiliations:** 1 Department of Internal Medicine, Rutgers New Jersey Medical School, Newark, NJ 07103, USA; 2 Division of Gastroenterology, Rutgers New Jersey Medical School, Newark, NJ 07103, USAaa2758@njms.rutgers.edu (A.A.-K.); 3 Division of Hepatology, Rutgers New Jersey Medical School, Newark, NJ 07103, USA; pjg47@njms.rutgers.edu

**Keywords:** metabolic dysfunction-associated steatotic liver disease (MASLD), resting heart rate, vibration-controlled transient elastography, hepatic steatosis, FIB-4, cardiometabolic dysfunction, noninvasive liver fibrosis assessment

## Abstract

*Background and Objectives*: Metabolic dysfunction-associated steatotic liver disease (MASLD) is closely linked to systemic cardiometabolic dysfunction. Resting heart rate (RHR), a marker of autonomic and metabolic stress, has been associated with metabolic syndrome and cardiovascular disease, though its relationship with MASLD remains incompletely characterized. We evaluated the association between RHR and liver-related outcomes in a nationally representative U.S. cohort. *Materials and Methods*: We analyzed adults aged 20–79 years from NHANES 2017–2020 with valid vibration-controlled transient elastography (VCTE) measurements. Participants with significant alcohol use, viral hepatitis, pregnancy, use of heart rate-modifying medications, or missing key data were excluded. Liver outcomes included elevated alanine aminotransferase (ALT), advanced steatosis (CAP ≥ 290 dB/m), FIB-4 fibrosis category, and VCTE-defined fibrosis. Multivariable regression models adjusted for demographic and metabolic covariates were used to evaluate associations between RHR and liver-related outcomes. *Results*: A total of 3532 adults were included. Each 1 beat-per-minute increase in RHR was independently associated with elevated ALT (OR 1.025, 95% CI 1.002–1.050, *p* = 0.043) and advanced steatosis (OR 1.036, 95% CI 1.001–1.073, *p* = 0.048). No significant association was observed between RHR and higher FIB-4 category (OR 1.025, 95% CI 0.992–1.060, *p* = 0.169) or VCTE-defined fibrosis (OR 0.990, 95% CI 0.961–1.021, *p* = 0.481). Associations with ALT and steatosis were attenuated after adjustment for glycemic and lipid parameters. *Conclusions*: Higher RHR was associated with steatosis-related and metabolic liver outcomes, but not fibrosis, in a nationally representative cohort. These findings suggest that RHR may reflect underlying autonomic and metabolic dysfunction relevant to early MASLD pathogenesis rather than advanced fibrotic disease.

## 1. Introduction

Metabolic dysfunction-associated steatotic liver disease (MASLD) is the most common cause of chronic liver disease worldwide, affecting approximately 25% of the global population and closely paralleling the rising prevalence of obesity and type 2 diabetes mellitus [1]. Its pathogenesis is driven by complex interactions between insulin resistance, systemic inflammation, and excess visceral adiposity, placing MASLD within the broader spectrum of cardiometabolic disease [2].

Resting heart rate (RHR) is a readily obtainable physiologic measure that reflects autonomic nervous system activity and overall cardiometabolic stress. Dysregulation of autonomic balance, characterized by increased sympathetic and reduced parasympathetic activity, has been associated with metabolic abnormalities and shown to predict the development of metabolic syndrome [3,4]. Additionally, higher RHR has been linked to impaired glucose regulation, incident metabolic syndrome, and vascular dysfunction, including arterial stiffness [5,6,7,8]. These metabolic disturbances share key pathophysiologic mechanisms with MASLD, including insulin resistance and chronic low-grade inflammation.

A growing body of literature highlights a strong association between MASLD and cardiovascular disease. Observational studies and meta-analyses have demonstrated that MASLD is associated with increased risks of heart failure and major adverse cardiovascular events [9,10,11]. Mechanistic and translational studies further indicate that MASLD may contribute to cardiac remodeling and arrhythmogenesis through pathways involving systemic inflammation, metabolic dysregulation, and autonomic dysfunction [12,13].

Despite these overlapping pathophysiologic pathways, relatively few studies have directly evaluated the relationship between RHR and MASLD. Existing evidence remains limited, with some studies demonstrating an association between elevated RHR and nonalcoholic fatty liver disease (NAFLD) in select populations [14] and incomplete adjustment for key confounders while lacking fibrosis outcomes [15]. We hypothesized that elevated RHR would be associated with MASLD-related liver abnormalities in a heterogeneous population due to the aforementioned shared metabolic pathways.

We analyzed data from the National Health and Nutrition Examination Survey (NHANES) 2017–2020 to evaluate the association between RHR and liver-related outcomes, including hepatic steatosis, liver enzyme elevation, and fibrosis. By incorporating vibration-controlled transient elastography (VCTE), FIB-4 scoring, and comprehensive adjustment for cardiometabolic factors, this study aims to determine whether RHR may serve as a simple, low-cost marker for identifying individuals at increased risk for MASLD. To our knowledge, this is the first study to evaluate the association between resting heart rate and MASLD-related liver outcomes in a large and nationally representative U.S. cohort.

## 2. Materials and Methods

### 2.1. Study Population and Design

We analyzed data in adults aged 20–79 years using NHANES 2017–2020 cycles that provided valid VCTE measurements. This time frame was specifically selected because it represents the only recent NHANES cycle that simultaneously includes elastography data and detailed medication variables necessary to reliably exclude medications that alter heart rate. NHANES was utilized as a data source as it employs a complex, multistage probability sampling design to generate nationally representative estimates of the non-institutionalized U.S. population.

**Figure 1:**
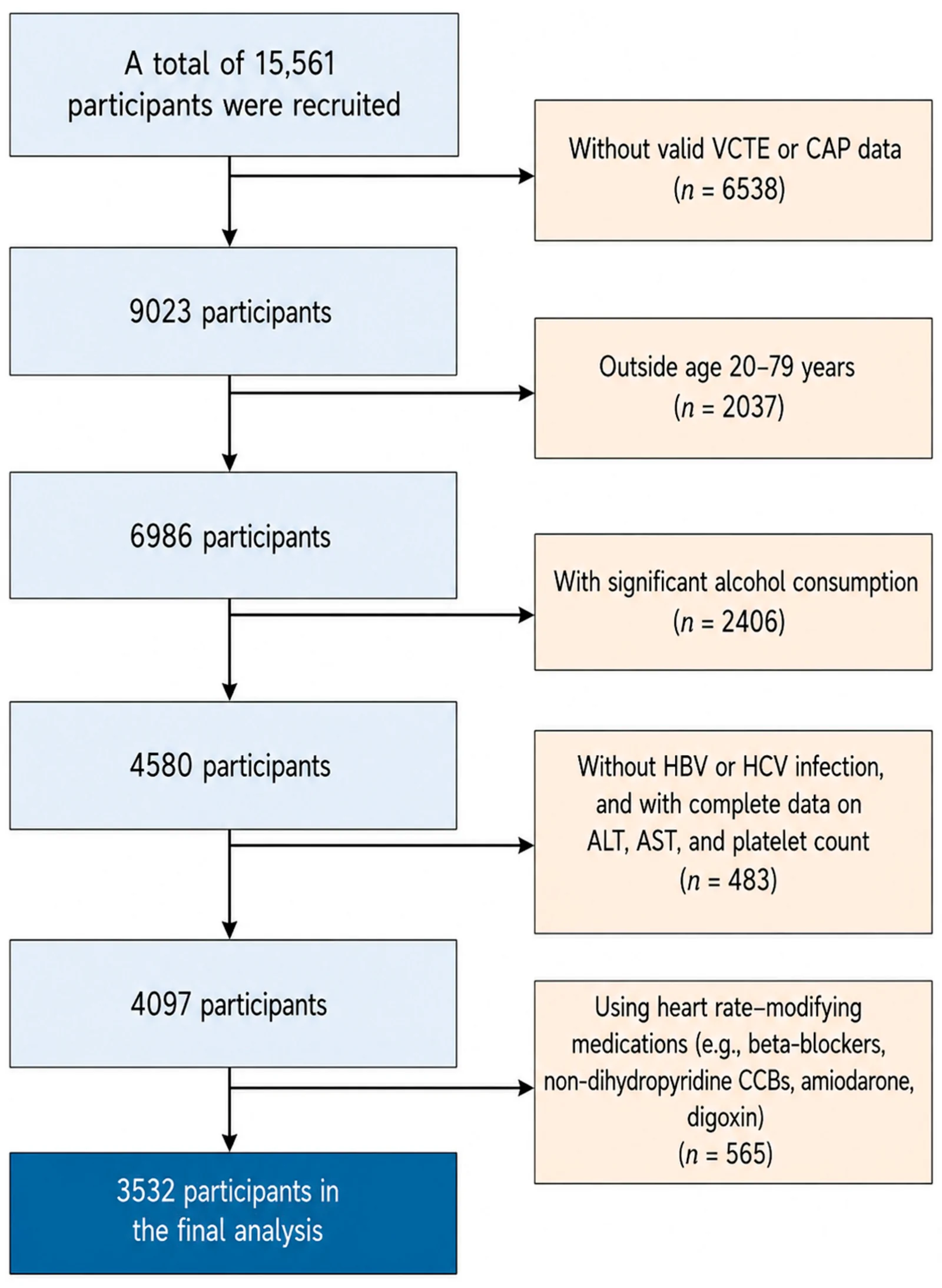
Participant selection process for inclusion in the study analysis.

Individuals were excluded if they were pregnant, had evidence of chronic viral hepatitis, reported significant alcohol use (>14 drinks/week for men; >7 drinks/week for women), were taking heart rate-modifying agents (beta-blockers, verapamil, diltiazem, amiodarone, sotalol, digoxin, dofetilide and disopyramide), or had missing laboratory or elastography data required for analysis. The participant inclusion and exclusion process is illustrated in Figure 1.

### 2.2. Exposure Variable

RHR was measured under standardized resting conditions in the NHANES Mobile Examination Center. Following 5 min of seated rest, three consecutive oscillometric blood pressure and pulse measurements were obtained at short intervals. RHR was calculated as the mean of the three recorded pulse measurements. RHR was analyzed as a continuous variable (per 1 beat per minute increment).

### 2.3. Outcome Definitions

Liver-related outcomes were defined as follows. Elevated alanine aminotransferase (ALT) was defined as >33 IU/L for men and >25 IU/L for women. Fibrosis risk was assessed using the FIB-4 index and categorized as low (<1.3), indeterminate (1.3–2.67), or high risk (>2.67) for study participants aged under 60. For participants 60 years of age and older at time of survey, FIB-4 index was categorized as low (<2.0), indeterminate (2.0–2.67), and high risk (>2.67). Hepatic steatosis was quantified using controlled attenuation parameter (CAP), with advanced steatosis defined as CAP ≥ 290 dB/m. Liver fibrosis severity was assessed using liver stiffness measurements (LSMs) from VCTE and categorized as <8 kPa, 8–12 kPa, and >12 kPa. All liver-related outcome thresholds and fibrosis risk categories were selected based on commonly utilized cutoffs supported by American Association for the Study of Liver Diseases (AASLD) guidance and the prior validated MASLD/VCTE literature [16].

### 2.4. Statistical Analysis

Multivariable logistic regression models were used to assess the association between continuous RHR and each liver-related outcome, adjusting for age, sex, body mass index (BMI), race/ethnicity, smoking status, and alcohol intake. For binary outcomes (elevated ALT and advanced steatosis), standard logistic regression was applied. For ordinal outcomes (FIB-4 category and fibrosis category), proportional odds logistic regression models were used, with the odds ratio interpreted as the odds of being in a higher-risk category per 1 bpm increase in RHR.

Effect modification was evaluated by including interaction terms between RHR and obesity status (BMI ≥ 30 kg/m^2^) and diabetes status (HbA1c ≥ 6.5%) in separate models. Statistically significant interactions were further explored through stratified analyses.

Sensitivity analyses were performed by sequentially adding individual cardiometabolic and inflammatory covariates to the primary multivariable models to evaluate the robustness of the observed associations. These covariates included ferritin, triglycerides, systolic blood pressure, C-reactive protein (CRP), high-density lipoprotein (HDL) cholesterol, hemoglobin A1c (HbA1c), and sedentary time.

Discriminative performance of RHR alone and in multivariable-adjusted models was assessed using the area under the receiver operating characteristic curve (AUC/C-statistic) for elevated ALT and advanced steatosis.

All analyses incorporated NHANES sample weights, primary sampling units, and strata to account for the complex survey design and to produce nationally representative estimates. Statistical analyses were conducted using SAS 9.4, and statistical significance was defined as a two-tailed *p*-value < 0.05.

## 3. Results

### 3.1. Study Population

A total of 3532 adults aged 20–79 years with valid VCTE were included in the final analysis. The mean age was 47.6 years, 53.8% were male, and the mean RHR was 69.8 beats/min. The cohort had a mean BMI of 29.3 kg/m^2^ and was racially and ethnically diverse. Baseline demographic and clinical characteristics are summarized in Table 1. This nationally representative sample provided a robust cohort to evaluate the association between RHR and liver-related outcomes.

### 3.2. Association Between Resting Heart Rate and Liver Outcomes

In multivariable logistic regression models adjusted for age, sex, BMI, race/ethnicity, smoking status, alcohol intake, and income-to-poverty ratio, higher RHR was independently associated with multiple liver-related outcomes.

**Figure 2:**
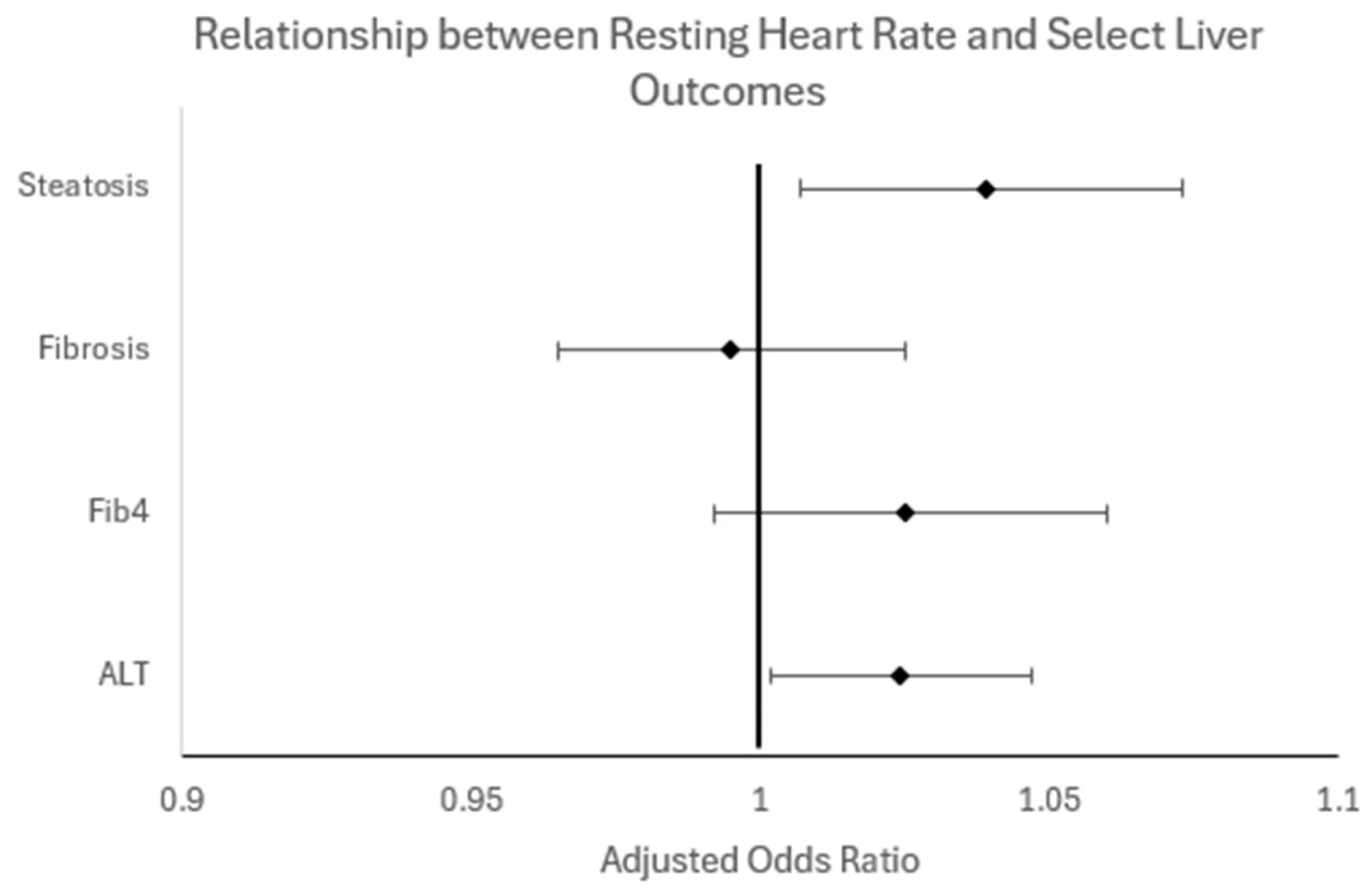
Adjusted odds ratios for the association between resting heart rate and liver outcomes.

Each 1 beat-per-minute increase in RHR was independently associated with elevated ALT (OR 1.025, 95% CI 1.002–1.050, *p* = 0.043) and advanced steatosis (OR 1.036, 95% CI 1.001–1.073, *p* = 0.048). No significant association was observed between RHR and higher FIB-4 category (OR 1.025, 95% CI 0.992–1.060, *p* = 0.169) or VCTE-defined fibrosis (OR 0.990, 95% CI 0.961–1.021, *p* = 0.481). Results are portrayed in Table 2 and in a forest plot in Figure 2.

### 3.3. Effect Modification

Effect modification analyses demonstrated significant interactions between RHR and both obesity and diabetes status. Obesity modified the association between RHR and elevated ALT, while diabetes modified the association with advanced steatosis. No significant interactions were observed for age, sex, or race/ethnicity.

In stratified analyses, the association between RHR and elevated ALT was more pronounced among individuals with obesity, while the association between RHR and advanced steatosis was stronger among individuals with diabetes (Table A1).

### 3.4. Sensitivity Analyses and Metabolic Covariate Adjustment

#### 3.4.1. FIB-4 Category

RHR was not associated with the liver stiffness-defined fibrosis category in the baseline model, and this null finding persisted across all adjusted models. Effect estimates remained small and non-significant regardless of covariate inclusion (Table 3).

#### 3.4.2. Fibrosis

RHR was not associated with liver stiffness-defined fibrosis in the baseline model, and this null finding persisted across all adjusted models. Effect estimates remained small and non-significant regardless of covariate inclusion (Table 3).

#### 3.4.3. Elevated ALT

The association between RHR and elevated ALT remained significant after adjustment for multiple cardiometabolic and inflammatory covariates including systolic blood pressure, C-reactive protein, and sedentary time (all *p* < 0.05). However, after adjustment for hemoglobin A1C, the association was attenuated and no longer statistically significant, with reversal in the direction of effect (AOR = 0.952, *p* = 0.157). Adjustment for triglycerides, ferritin and HDL cholesterol also attenuated the association, though the direction of effect remained positive (Table 3).

#### 3.4.4. Steatosis

Similarly, the association between RHR and hepatic steatosis remained significant after adjustment for several cardiometabolic variables, including ferritin, systolic blood pressure, and sedentary time. However, adjustment for hemoglobin A1C attenuated the association and resulted in loss of statistical significance (AOR = 0.963, *p* = 0.2735), with reversal in direction. Adjustment for triglycerides, C-reactive protein and HDL cholesterol also attenuated the association, though the direction of effect remained positive (Table 3).

### 3.5. Discriminative Performance

**Figure 3:**
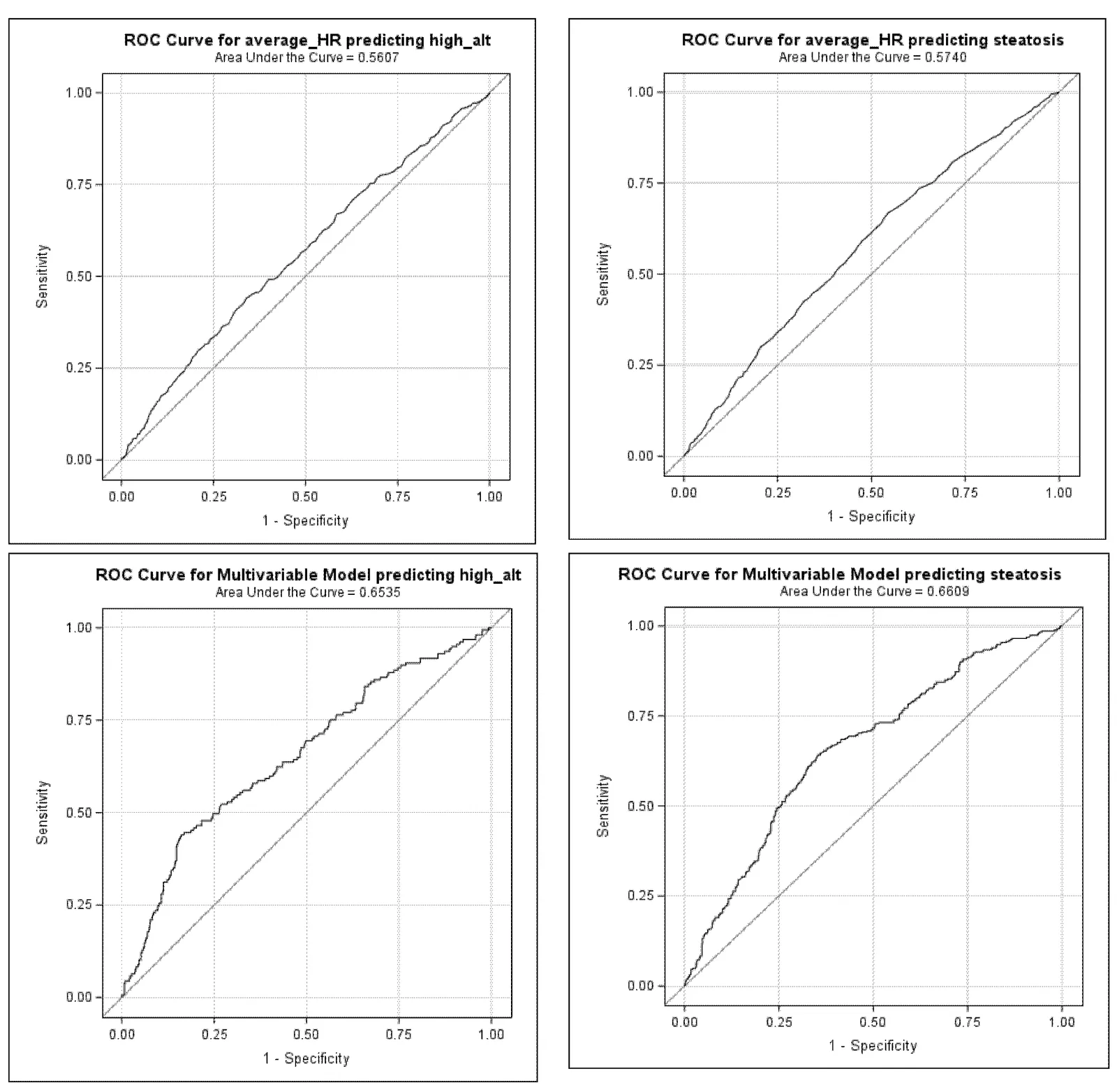
Receiver Operating Characteristic (ROC) Curves for Resting Heart Rate and Multivariable Models Predicting Elevated ALT and Advanced Steatosis.

RHR alone demonstrated limited discriminative ability for elevated ALT and advanced steatosis, with area under the receiver operating characteristic curve (AUC) values of 0.56 and 0.57, respectively (Figure 3).

In contrast, multivariable models incorporating RHR alongside established clinical and metabolic covariates demonstrated improved discrimination, with AUC values of 0.65 for elevated ALT and 0.66 for advanced steatosis (Figure 3). These findings indicate that while RHR alone has modest predictive utility, it may provide modest incremental value when incorporated into multivariable risk models.

## 4. Discussion

In this nationally representative analysis of U.S. adults, higher RHR was independently associated with elevated ALT and advanced hepatic steatosis, but was not significantly associated with either higher FIB-4 category or liver stiffness-defined fibrosis. These results propose that RHR is more closely linked to metabolically active liver disease, particularly steatosis and biochemical liver injury, rather than fibrosis risk. Associations with ALT and steatosis were attenuated after adjustment for hemoglobin A1C and lipid parameters, suggesting that the association is largely driven by underlying metabolic dysfunction. The observed effect modification by obesity and diabetes further supports this interpretation.

These results both align with and extend the prior literature. Kim and Lee demonstrated an independent association between higher RHR and NAFLD in post-menopausal women, proposing autonomic imbalance, insulin resistance, and inflammation as potential mechanisms [14]. In a UK Biobank study, Jamialahmadi et al. showed that higher RHR was associated not only with fatty liver disease but also with cardiac remodeling phenotypes, supporting the concept that RHR reflects broader cardiometabolic dysfunction rather than isolated hepatic pathology [15]. Unlike these prior studies relying on specific populations, narrow imaging or limited covariate adjustment, our analysis incorporates VCTE and demonstrates that this association is most evident for steatosis-related and metabolic markers, while not extending to fibrosis as assessed by either FIB-4 or VCTE.

A key insight from our results is the agreement between FIB-4 and elastography-based fibrosis outcomes; RHR was not associated with higher FIB-4 category nor liver stiffness-defined fibrosis. This denotes that RHR is more closely tied to earlier and metabolically active stages of MASLD, such as hepatic steatosis and hepatocellular injury, rather than established fibrosis. Elevated RHR reflects increased sympathetic activity and reduced parasympathetic tone, which can contribute to insulin resistance through increased hepatic glucose production, enhanced lipolysis, and increased free fatty acid delivery to the liver [12,14]. These overlapping metabolic pathways may explain the attenuation seen after adjustment for A1C and lipid parameters. By contrast, adjustment for hemoglobin produced minimal changes in the effect estimates, suggesting that anemia-related physiologic variation is unlikely to account for the observed association. Long-standing glycemic impairment may contribute to cardiac autonomic neuropathy, which is often characterized by sympathetic predominance and resting tachycardia in its early stages [17]. Thus, elevated RHR in individuals with MASLD may partially reflect underlying autonomic dysfunction, providing an additional physiologic link between metabolic dysregulation and hepatic steatosis. In contrast, fibrosis represents a chronic and cumulative process involving stellate cell activation, extracellular matrix deposition, and architectural remodeling that may not be reflected by a single physiologic parameter in a cross-sectional analysis [12].

More broadly, these results reflect the close relationship between MASLD and cardiovascular-metabolic disease. MASLD is increasingly recognized as a multisystem disorder linking hepatic metabolic dysfunction with cardiovascular remodeling. Emerging evidence has also demonstrated a strong association between MASLD and heart failure with preserved ejection fraction (HFpEF), with shared underlying mechanisms including systemic inflammation, insulin resistance, endothelial dysfunction, and myocardial remodeling [18]. Insulin resistance, sympathetic activation, and renin–angiotensin–aldosterone system activity contribute to both hepatic steatosis and cardiac remodeling, while excess fatty acid flux, lipotoxic intermediates, and oxidative stress promote myocardial dysfunction. In parallel, hepatic lipid accumulation drives systemic inflammation and release of cytokines and other mediators that contribute to cardiac remodeling, electrical instability, and autonomic dysfunction [12,13,15]. Together, these pathways help explain why RHR may reflect an integrated state of metabolic stress linking hepatic steatosis with more widespread cardiometabolic disease.

The observed effect modification by obesity and diabetes is consistent with this systemic model. MASLD is highly prevalent in these populations, and prior studies have shown that the cardiovascular consequences of NAFLD/MASLD are more pronounced in individuals with diabetes [15]. In our study, obesity amplified the association between RHR and ALT, while diabetes strengthened the association with steatosis, proposing that RHR is most informative in metabolically high-risk individuals.

From a clinical perspective, these findings align with current MASLD screening strategies, which emphasize identification of at-risk individuals followed by stepwise risk stratification using noninvasive tests such as FIB-4 and elastography [16,19]. RHR is unlikely to serve as a standalone screening tool given its limited discriminative ability; however, its consistent association with metabolic liver disease suggests that it may function as a simple, low-cost adjunctive signal prompting further evaluation, particularly in individuals with obesity or diabetes. Elevated RHR may help identify metabolically high-risk individuals who warrant further evaluation and risk-factor modification.

This study has several strengths, including the use of a large, nationally representative dataset, elastography-based assessment of liver disease, and comprehensive evaluation across multiple liver-related outcomes. The inclusion of detailed metabolic covariates and effect modification analyses allows for more nuanced interpretation of the physiologic relationships underlying these associations. The application of age-adjusted FIB-4 thresholds further strengthens the fibrosis analysis by accounting for the known influence of age on FIB-4 classification.

However, several limitations should be considered. The cross-sectional design limits causal inference, and RHR is influenced by multiple non-hepatic factors, including medications, fitness level, and comorbid conditions. Additionally, the broad age range of the study population may have introduced heterogeneity in factors affecting resting heart rate, including differences in comorbidities, medication use, physical conditioning, and autonomic function. To help mitigate these effects, our analyses adjusted for age and multiple demographic and metabolic covariates, and age-adjusted FIB-4 thresholds were applied in fibrosis risk stratification. Moreover, thyroid function data were not available in NHANES 2017–March 2020 and therefore could not be included in our analyses. Although unmeasured thyroid disease may have contributed to residual confounding, hypothyroidism is more prevalent than hyperthyroidism and is associated with both bradycardia and MASLD [20], suggesting that any resulting bias would more likely attenuate rather than strengthen the observed associations. Furthermore, the modest discriminative performance of resting heart rate alone indicates that it should not be interpreted as a standalone screening tool but rather as an exploratory physiologic marker that may complement established clinical risk assessment. Finally, the null findings for FIB-4 and VCTE-defined fibrosis should be interpreted in the context of a general population cohort, where advanced fibrosis prevalence may be relatively low and longitudinal progression cannot be assessed.

Future studies should evaluate whether elevated RHR identifies patients with MASLD who are more likely to develop worsening metabolic disease, cardiovascular complications, or progression of liver injury over time. This may be particularly important in patients with obesity or diabetes, where the relationship between RHR and liver outcomes appeared strongest in our analysis. Incorporating repeated RHR measurements alongside longitudinal liver assessments may help determine whether RHR is simply a marker of baseline metabolic risk or a useful signal of ongoing cardiometabolic and hepatic disease activity.

## 5. Conclusions

In conclusion, higher RHR was associated with steatosis-related and metabolic liver outcomes, including elevated ALT and advanced hepatic steatosis, but not with fibrosis as assessed by either FIB-4 or VCTE in a nationally representative cohort. These findings support the concept that RHR reflects underlying autonomic and metabolic dysregulation relevant to early MASLD pathogenesis rather than advanced fibrotic disease. The attenuation of these associations after adjustment for glycemic and lipid parameters further suggests that RHR primarily reflects broader cardiometabolic dysfunction associated with MASLD, rather than serving as a liver-specific biomarker. Although RHR demonstrated limited standalone predictive utility, it may serve as a simple adjunctive physiologic signal prompting further metabolic and liver risk assessment in high-risk populations. Future longitudinal studies may help clarify whether elevated RHR is a marker of early MASLD development or progressive metabolic liver dysfunction.

## Figures and Tables

**Table 1:** Baseline demographic and clinical characteristics of included participants.

Participant Baseline Characteristics	Mean (SD) or %
Average Heart rate	69.8 (0.4)
Age	47.6 (0.5)
Male Sex	53.8%
BMI	29.3 (0.3)
Self-Identified Race/Hispanic Origin	
Mexican American	8.1%
Other Hispanic	7.8%
Non-Hispanic White	59.9%
Non-Hispanic Black	11.5%
Non-Hispanic Asian	8.8%
Other Race—Including Multi-racial	4.0%
Smoking Status	
Never smoker	65.5%
Former smoker	24.0%
Current smoker	10.5%
Alcohol Intake (past 12 months)	
None in past year	34.0%
1–11 times/year	24.1%
1–3 times/month	18.8%
1–4 times/week	18.5%
Every/nearly every day	4.6%
Diabetes Status	
Yes	9.7%
No	87.5%
Borderline	2.7%

Included in dataset after exclusion criteria applied. Continuous characteristics are weighted means with standard errors shown in parentheses; categorical rows show weighted percentages among non-missing responses (smoking *n* = 3531; alcohol *n* = 3273; diabetes *n* = 3530).

**Table 2:** Adjusted odds ratios, 95% confidence intervals, and *p*-values for the association between resting heart rate and liver outcomes.

Outcome	Odds Ratio (95% CI)	*p*-Value	Standard Error
**Elevated ALT**	**1.02 (1.00, 1.05)**	**0.0426**	**0.0115**
FIB4	1.02 (0.99, 1.06)	0.1689	0.0168
Fibrosis	0.99 (0.96, 1.02)	0.4813	0.0146
**Steatosis**	**1.04 (1.00, 1.07)**	**0.0474**	**0.0168**

Odds ratios are for average heart rate from the primary adjusted models. Bold signifies statistical significance with *p*-value < 0.05.

**Table 3:** Odds ratio results of sensitivity analysis.

Additional Covariate	Odds Ratio	95% CI	*p*-Value
**Elevated ALT**			
Baseline	**1.025**	**1.001–1.050**	**0.0426**
Ferritin	1.024	0.999–1.049	0.0596
Triglycerides	1.022	1.000–1.045	0.0506
Systolic Blood Pressure	**1.026**	**1.001–1.052**	**0.0416**
C-Reactive Protein	**1.027**	**1.003–1.052**	**0.0285**
HDL Cholesterol	1.023	0.999–1.047	0.0626
HbA1c (Last A1C)	0.943	0.881–1.008	0.0817
Sedentary Minutes	**1.025**	**1.000–1.050**	**0.0490**
Hemoglobin	1.023	0.996–1.041	0.0750
**FIB-4 Category**			
Baseline	1.024	0.989–1.060	0.1689
Ferritin	1.025	0.991–1.061	0.1479
Triglycerides	1.026	0.991–1.061	0.1407
Systolic Blood Pressure	1.024	0.989–1.060	0.1696
C-Reactive Protein	1.024	0.988–1.061	0.1831
HDL Cholesterol	1.024	0.988–1.062	0.1865
HbA1c (Last A1C)	1.054	0.990–1.122	0.0925
Sedentary Minutes	1.024	0.989–1.061	0.1650
Hemoglobin	1.026	0.990–1.063	0.1451
**Liver Fibrosis**			
Baseline	0.990	0.960–1.020	0.4813
Ferritin	0.990	0.961–1.021	0.5129
Triglycerides	0.990	0.960–1.021	0.5049
Systolic Blood Pressure	0.989	0.959–1.019	0.4437
C-Reactive Protein	0.989	0.958–1.020	0.4672
HDL Cholesterol	0.991	0.962–1.021	0.5313
HbA1c (Last A1C)	0.980	0.924–1.040	0.4841
Sedentary Minutes	0.990	0.960–1.020	0.4907
Hemoglobin	0.991	0.961–1.021	0.5264
**Hepatic Steatosis**			
Baseline	**1.036**	**1.000–1.073**	**0.0474**
Ferritin	**1.036**	**1.000–1.073**	**0.0491**
Triglycerides	1.027	0.992–1.064	0.1243
Systolic Blood Pressure	**1.038**	**1.004–1.074**	**0.0294**
C-Reactive Protein	1.034	0.998–1.072	0.0645
HDL Cholesterol	1.032	0.992–1.073	0.1138
HbA1c (Last A1C)	0.962	0.897–1.033	0.2685
Sedentary Minutes	**1.033**	**1.001–1.066**	**0.0452**
Hemoglobin	**1.037**	**1.001 – 1.073**	**0.0445**

Note: Odds ratios and 95% confidence intervals estimated via logistic regression for average heart rate (per 1 bpm). Bold values indicate statistical significance (*p* < 0.05). CI = Confidence Interval. Each row represents a model additionally adjusted for the listed covariate beyond the baseline covariates. Bold signifies statistical significance with *p*-value < 0.05.

## Data Availability

The original data presented in the study are openly available in the National Health and Nutrition Examination Survey (NHANES) 2017–2020 database at https://wwwn.cdc.gov/nchs/nhanes/continuousnhanes/default.aspx?Cycle=2017-2020 (accessed on 10 November 2025).

## Abbreviations

HbA1c	Hemoglobin A1C
AASLD	American Association for the Study of Liver Diseases
ALT	Alanine Aminotransferase
AUC	Area Under the Curve
BMI	Body Mass Index
CAP	Controlled Attenuation Parameter
CI	Confidence Interval
ECG	Electrocardiogram
FFA	Free Fatty Acid
FIB-4	Fibrosis-4 Index
HDL	High-Density Lipoprotein
HFpEF	Heart Failure with Preserved Ejection Fraction
IR	Insulin Resistance
kPa	Kilopascal
LSM	Liver Stiffness Measurement
MASLD	Metabolic Dysfunction–Associated Steatotic Liver Disease
NAFLD	Nonalcoholic Fatty Liver Disease
NHANES	National Health and Nutrition Examination Survey
OR	Odds Ratio
RAAS	Renin–Angiotensin–Aldosterone System
RHR	Resting Heart Rate
ROC	Receiver Operating Characteristic
SNS	Sympathetic Nervous System
VCTE	Vibration-Controlled Transient Elastography

## Appendix A

**Table A1:** Stratified Associations Between Resting Heart Rate and MASLD Outcomes by Obesity and Diabetes Status.

	**Participants without Obesity**			**Participants with Obesity**		
	**AOR**	**95% CI**	***p*-Value**	**AOR**	**95% CI**	***p*-Value**
ALT	1.001	(0.974,1.029)	0.9179	1.048	(1.008,1.089)	0.0194
Fib-4 Category	1.052	(1.006,1.100)	0.0291	1.007	(0.957,1.060)	0.7688
Liver Fibrosis	1.001	(0.961,1.044)	0.9511	0.990	(0.955,1.026)	0.5629
Steatosis	1.035	(1.003,1.067)	0.0344	1.053	(1.002,1.107)	0.0426
	**Pa** **rticipants without Diabetes**			**Participants with Diabetes**		
	**AOR**	**95% CI**	***p*** * * **-Value**	**AOR**	**95% CI**	***p*** * * **-Value**
ALT	1.041	(1.017,1.065)	0.0014	0.982	(0.935,1.032)	0.4650
Fib-4 Category	1.034	(0.995,1.075)	0.0816	1.046	(0.980,1.116)	0.1672
Liver Fibrosis	1.008	(0.962,1.057)	0.7290	0.983	(0.939,1.030)	0.4578
Steatosis	1.050	(1.017,1.085)	0.0042	0.982	(0.935,1.031)	0.4528
